# An analysis of the positional distribution of DNA motifs in promoter regions and its biological relevance

**DOI:** 10.1186/1471-2105-9-89

**Published:** 2008-02-07

**Authors:** Ana C Casimiro, Susana Vinga, Ana T Freitas, Arlindo L Oliveira

**Affiliations:** 1 INESC-ID/IST, Rua Alves Redol, 9 1000-029 Lisboa, Portugal; 2 FCM/UNL, Campo dos Mártires da Pátria 130, 1169-056 Lisboa, Portugal

## Abstract

**Background:**

Motif finding algorithms have developed in their ability to use computationally efficient methods to detect patterns in biological sequences. However the posterior classification of the output still suffers from some limitations, which makes it difficult to assess the biological significance of the motifs found. Previous work has highlighted the existence of positional bias of motifs in the DNA sequences, which might indicate not only that the pattern is important, but also provide hints of the positions where these patterns occur preferentially.

**Results:**

We propose to integrate position uniformity tests and over-representation tests to improve the accuracy of the classification of motifs. Using artificial data, we have compared three different statistical tests (Chi-Square, Kolmogorov-Smirnov and a Chi-Square bootstrap) to assess whether a given motif occurs uniformly in the promoter region of a gene. Using the test that performed better in this dataset, we proceeded to study the positional distribution of several well known cis-regulatory elements, in the promoter sequences of different organisms (*S. cerevisiae*, *H. sapiens*, *D. melanogaster*, *E. coli *and several Dicotyledons plants). The results show that position conservation is relevant for the transcriptional machinery.

**Conclusion:**

We conclude that many biologically relevant motifs appear heterogeneously distributed in the promoter region of genes, and therefore, that non-uniformity is a good indicator of biological relevance and can be used to complement over-representation tests commonly used. In this article we present the results obtained for the *S. cerevisiae *data sets.

## Background

The computational analysis of DNA sequences represents a major endeavor in the post-genomic era. The increasing number of whole-genome sequencing projects has provided an enormous amount of information which leads to the need of new tools and string processing algorithms to analyze and classify the obtained sequences [1].

In this regard, the study of short functional DNA segments, such as transcriptional factor binding sites, has emerged as an important effort to understand key control mechanisms. For example, it is now known that the presence of certain sequences of motifs in promoter regions determines the effective regulation of gene transcription, a central feature of gene regulatory networks.

DNA motifs can be represented in a number of different ways. Position specific scoring matrices (PSSMs) and consensi (oligonucleotide sequences) are amongst the most commonly used. However, several other more sophisticated methods have been proposed to represent motifs, some of them able to take into account statistical or deterministic dependencies between positions [2]. Our approach is independent of the way motifs are modeled, since it requires only the list of occurrences of motifs, something that can be obtained from any motif representation.

Motif finding is the problem of discovering motifs, that may correspond to transcription factor binding sites, without any prior knowledge of their characteristics. These motifs can be found by analyzing regulatory regions taken from genes of the same organism or from related genes of different organisms. Many approaches have been proposed and one can find an impressive collection of published articles describing algorithms to address the problem.

Currently available methods can roughly be classified in two main classes: probabilistic [3,4] and combinatorial [5,6]. This classification covers most, although not all, popular motif finders currently available.

The major drawback with these algorithms is their inability to discriminate the biologically relevant extracted motifs from the potentially numerous false hits. Probabilistic motif finders also have problems when the motifs are highly degenerated. The problem of determining what portion of the output corresponds to a biologically significant result has been addressed mostly through the use of statistical techniques and biological reasoning, and it is a challenge in its own right. In this regard, the correct assessment of which of those observations may have occurred just by chance is a mandatory step in the process of identifying biologically meaningful features.

This is the main rationale for the construction of stochastic models that can provide estimates for the expected number of occurrences of a given sequence. These models are based on some assumed distribution for the sequence of bases, such as the one defined by a Markov chain [7], and are then used to compute the expected number of occurrences, under the null hypothesis, *H*_0_, that assumes that the sequence is randomly generated in accordance with the assumed distribution. Sequences that are over-represented, in a statistically significant way, are considered as potentially significant, as they are highly unlikely to have been generated by chance. This is usually done by determining a p-value for each extracted motif that assesses its relative expectedness/randomness under the specific pre-defined model, based on the expected vs. observed number of occurrences. This step makes for an efficient filtering of the output without loosing a significant amount of information and to the correct assessment of the motifs that are under or over-expressed. However, this approach is not very selective, and it is hard to apply to small motifs (that occur very frequently by chance) or to other motifs that are not over-represented, but that, nonetheless, are biologically significant.

One possible way around this limitation is to look at other characteristics of the motifs, such as the positional distribution in the regions under analysis. The idea of analyzing the positional distribution of motif occurrences was developed recently [8,9], suggesting that several motifs occurring in natural sequences have strong positional preferences. For example, it is well known that in prokaryote promoter regions, the TATA-box occurs near position -10 (before the beginning of transcription) and the TTGACA motif usually occurs near position -35. In eukaryotes, the best-characterized core promoter elements consist of a TATA-box located approximately 30 nucleotides upstream from the start site, an initiator element located at the transcription start site, and a downstream promoter element (DPE) located approximately 30 nucleotides downstream from the transcription start.

The functionality of genome regions is intrinsically related with their ability to fold in tri-dimensional structures, which clearly indicates that positional bias should be incorporated in the models and analyzed from a statistical point of view. A number of recent studies have focused on this property, both in terms of absolute [10-12] and relative [13,14] positioning of the different motifs. These studies, however, address the positioning of specific, well identified, motifs, but none of them, to our knowledge, presented complete quantitative results and a comprehensive analysis of this feature, that can be used to actually distinguish between relevant and non-relevant motifs. In particular, there is no clear proposal of which is the best test to identify and classify the motifs in terms of their positional distribution along the genome.

In this article we propose to integrate position uniformity tests and over-representation tests based on Markov models to improve the posterior classification of the motifs and better assess their biological significance.

For the position uniformity tests, the input data corresponds to vectors of motif positions in the input sequences where the motif appears. Specifically, we compute the position of each occurrence of each motif, relative to the translation start site, and build a list of these positions. This list will be analyzed for uniformity using a statistical test. We started this analysis by first comparing different statistical methodologies commonly used to test uniformity, namely the Chi-Square goodness-of-fit test and the Kolmogorov-Smirnov (KS) test [8]. Since the motifs may appear in a small number of positions, a bootstrap [15] version of the Chi-Square test, that can cope with small sample sizes, was also evaluated. These tests were first validated on artificially generated data to better analyze the results, assessing their sensitivity and specificity as well as the Receiver Operating Characteristic (ROC) curves. Based on these results, the bootstrap Chi-Square test was chosen as it proved to be the most powerful test for small sample sizes.

The bootstrap Chi-Square test was then used in the study of the positional distribution of motifs in the promoter sequences of different organisms, namely bakers yeast (*Saccharomyces cerevisiae*), human (*Homo sapiens*), fruit fly (*Drosophila melanogaster*), *Escherichia coli *and several Dicotyledons plants. Motifs that appeared at least in a given fraction of the sequences (the quorum) were extracted, from these data sets, using a motif finder. For each extracted motif, a p-value was calculated, under the null hypothesis, that assumes a uniform distribution for the motif positions.

The combination of over-representation tests, that take into account the specific sequence of bases, and position uniformity tests, that consider the positional distribution of the motifs, has the potential to represent a more powerful technique for motif classification, than the ones currently used. This methodology is easily adapted to other computational biology applications where position is thought to be biologically relevant and where the non-uniformity of observed motifs may provide strong indication of biological relevance (e.g., [10]).

## Results and discussion

In this section we present the results of applying the proposed methodology to both artificially generated (synthetic) and real data sets.

The artificial data sets were first used to compare the three analyzed uniformity tests, the Chi-Square goodness-of-fit test, the Kolmogorov-Smirnov test and the Chi-Square bootstrap test. The uniformity test that obtained the best results was then applied to classify the motifs extracted, by a motif finder, from a number of real data sets, from different organisms. In this section we present only the results obtained for the *Saccharomyces cerevisiae *data sets, but results for other organisms are also available [see Additional file 1].

### Comparison of Position Uniformity Tests

Artificial data sets generated from uniform and beta distributions were used in this comparison (see section Methods). Specifically, we generated a number of positions, normalized to the unit interval, that emulate, what, in the real data, corresponds to the positions of motif occurrences. For each of these distributions, the sensitivity and the specificity of the three uniformity tests was assessed. Specifically, we used these tests to evaluate the probability that *H*_0 _is true, i.e., that the given samples originated in a uniform distribution.

**Figure 1:**
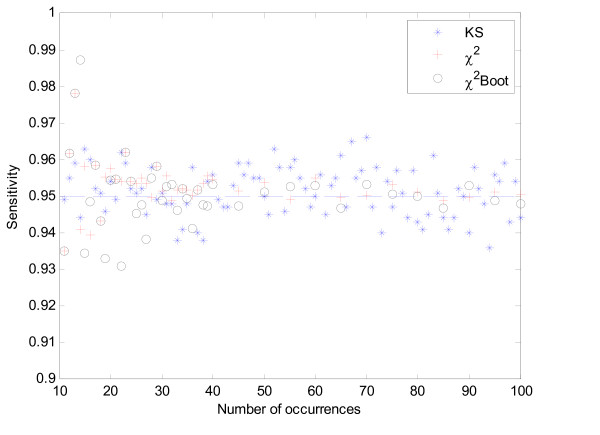
Sensitivity comparison for the Chi-Square, Kolmogorov-Smirnov and bootstrap Chi-Square test as a function of the number of occurrences, under a uniform distribution of positions.

**Figure 2:**
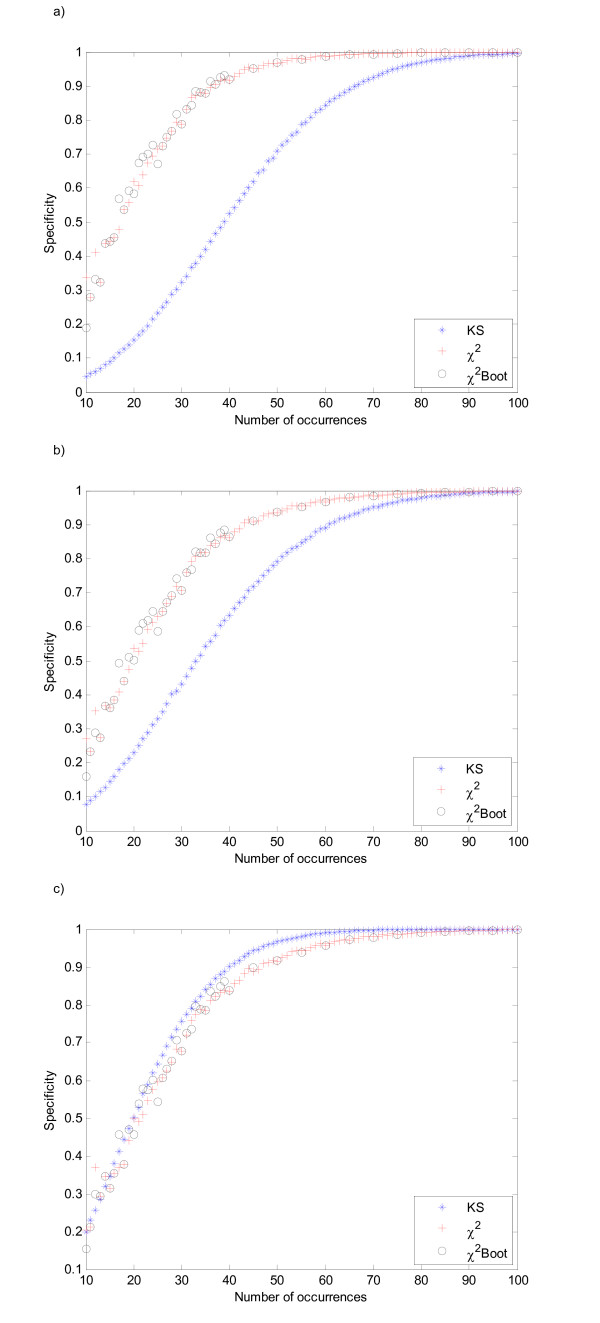
Specificity comparison for the Chi-Square, Kolmogorov-Smirnov and bootstrap Chi-Square test as a function of the number of occurrences, under the Beta distribution, for parameters a) (3, 3), b) (15, 15) and c) (3, 1.5).

**Figure 3:**
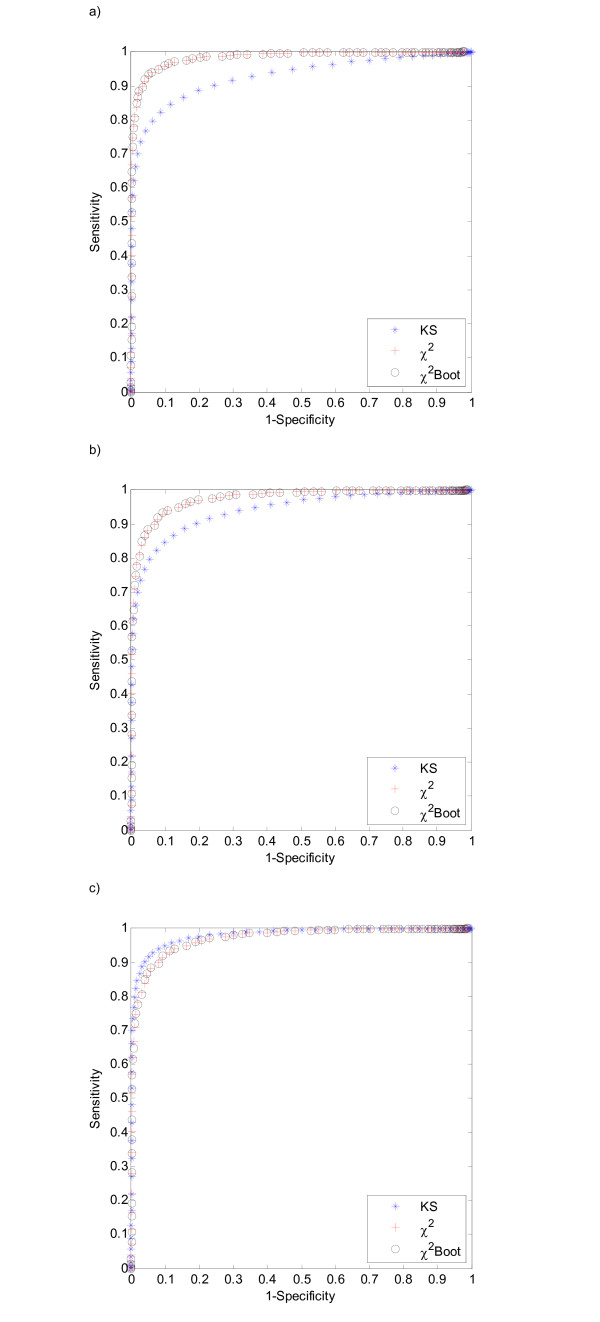
ROC curves for Chi-Square, Kolmogorov-Smirnov and bootstrap Chi-Square tests. Uniform and Beta samples with 40 points each were used. a) Beta(3,3), b) Beta(15,15) and c) Beta(3,15).

The results obtained are presented in Figures 1 and (2a, b and 2c). Figure (3a, b and 3c) illustrate the obtained ROC curves, as described in the Methods section, for distinct Beta distribution functions, Beta(3,3), Beta(3,2.5) and Beta(3,2), respectively. These curves were calculated using a sample size of 40 observations and 8 bins to guarantee five expected counts per class.

The results show that all tests present the expected sensitivity, as imposed by the level of significance (in this case, it represents the probability that a uniform sample will be classified as non-uniform). They correctly identified the majority, around 95%, of the uniform instances of different lengths when a level of *α *= 5% is chosen.

Instances drawn from Beta distributions were in general also correctly identified by the three tests. The more asymmetric the distribution is, the more powerful the KS test becomes. For distributions with lower variance and higher symmetry the bootstrap version of Chi-Square becomes the more powerful test, as shown in Figure 2(a).

The power of the tests increases as the samples become larger, as would be expected. From these results it is possible to see that the Kolmogorov-Smirnov test is less powerful than the other two tests. Both Chi-Square goodness-of-fit and the Chi-Square bootstrap tests present a similar behavior.

For small sample sizes, the determination of the optimal number of bins in the Chi-square test was achieved by testing different rules. One of the most common is to guarantee a minimum number of five expected counts per bin, which leads to *nbins *= ⌊*x*/5⌋, where *x *is the sample size. However, when this value is between 10 and 15 it is advisable to use three classes. In effect, it would be virtually impossible to distinguish a symmetric distribution using two bins defined by the edges (0, 0.5, 1). When using three bins there is a significant increase of specificity values, which strengthens the application of Chi-square with a modified rule for the number of bins. Furthermore, this assessment and further testing on simulated data led to the application of a modified rule for determining the number of bins, described by *nbins *= ⌊*x*/5⌋ + 1, for sample sizes up to 40. This was shown to increase the specificity. The graphs of Figure 2 show the specificity of the test for different Beta distribution functions as a function of sample size, using a combination of both rules: from 10 to 40 one extra bin was applied to improve the classification, from 40 to 100 the usual 5-per-bin formula was employed.

These results also show that there is a real advantage in using bootstrap methodologies. When the size of the sample is small, (*N *< 30), the results show that the Chi-Square bootstrap test exhibits the best ability to detect non-uniformity. Although the difference between the bootstrap version and the standard test appears to be marginal, one should use the simulation results to fix the desired sensitivity. In fact, only by using the empirical distribution of the test statistic, is it possible to correctly adjust the level of significance for small samples. This is because the distribution in this case markedly deviates from the Chi-Square standard distribution.

These results therefore indicate that the Chi-Square test with bootstrap achieves the best performance and should therefore be used to assess the uniformity of motif positions in real data, specially when the number of occurrences is small.

### Analysis of the *Saccharomyces cerevisiae *data sets

In this section we present the results obtained with the Chi-Square bootstrap test in the analysis of the positional distribution of DNA motifs in the promoter regions of *Saccharomyces cerevisiae*. For this analysis, six data sets of promoter sequences were considered (see section Methods). For each data set the following procedure was used:

1. A motif finder algorithm, RISO [5], was used to identify motifs that occurred in a given fraction (the quorum) of the sequences.

2. Motifs were ordered in accordance to the computed statistical significance of the deviation of the number of occurrences observed vs. expected. Motifs were considered over-represented in a statistically significant way if the p-value was smaller than 10^-3^.

3. A p-value for the likelihood of a uniform distribution of each motif was obtained, using the Chi-Square bootstrap test, using a significance level, *α*, equal to 0.05.

With this analysis, it is possible to define four motif classes, according to their classification in the over-representation and uniformity tests. Motifs that are both over-represented and non-uniformly distributed are very likely to have some biological function. Our analysis will be centered on these motifs, although, at times, we will also look at motifs that are not seen as strongly over-represented but are distributed in a non-uniform way in the promoter regions.

#### Core promoter elements

For the analysis of the positional distribution of several core promoter elements, a data set containing 5864 promoter sequences from *Saccharomyces cerevisiae *was considered. This data set includes a significant fraction of the gene promoters for this organism. For eukaryotic species there exists a set of well characterized core promoter elements, that include the *TATA-box *and the *GC-box*. The documented consensi for these sites are:

• *TATA-box *[16]:

$$\begin{array}{cccccccc} \text{T} & \text{A} & \text{T} & \text{A} & \left(\text{A}/\text{T}\right) & \text{A} & \left(\text{A}/\text{T}\right) & \left(\text{A}/\text{G}\right) \end{array}$$

• *GC-box*: [17]

$$\begin{array}{llllllll} \text{g} & \text{G} & \text{G} & \text{G} & \text{C} & \text{G} & \text{G} & \text{g}\\ \text{t} & \text{a} & & & \text{t} & \text{a} & \text{t} & \text{a}\\ \text{a} & & & & \text{a} & & & \text{t} \end{array}$$

Following the procedure described, the RISO algorithm was instructed to extract simple motifs, with sizes between 5 and 8, and a minimum quorum of 20%. Table 1 shows the results obtained. It is possible to see that almost all the motifs extracted (2640 out of 2924) were classified as being non-uniformly distributed.

**Table 1:** Distribution of motifs according to uniformity and statistical significance in the global *S. cerevisiae *data set.

Motifs	Uniform	Non uniform	Total
Not over-represented	254	2002	2256
Over-represented	30	638	668

Total	284	2640	2924

From the non-uniform group, 638 were classified as over-represented in a statistically significant way. The first ranked motifs in this sub-group correspond to motifs that match previously described consensi. For example, motifs like TATAAA, TATATA, TATATAA, TATATAT, and TATAAAA match with the *TATA-box*; GGGTA, and GGGCG fit the *GC-box *profile.

**Figure 4:**
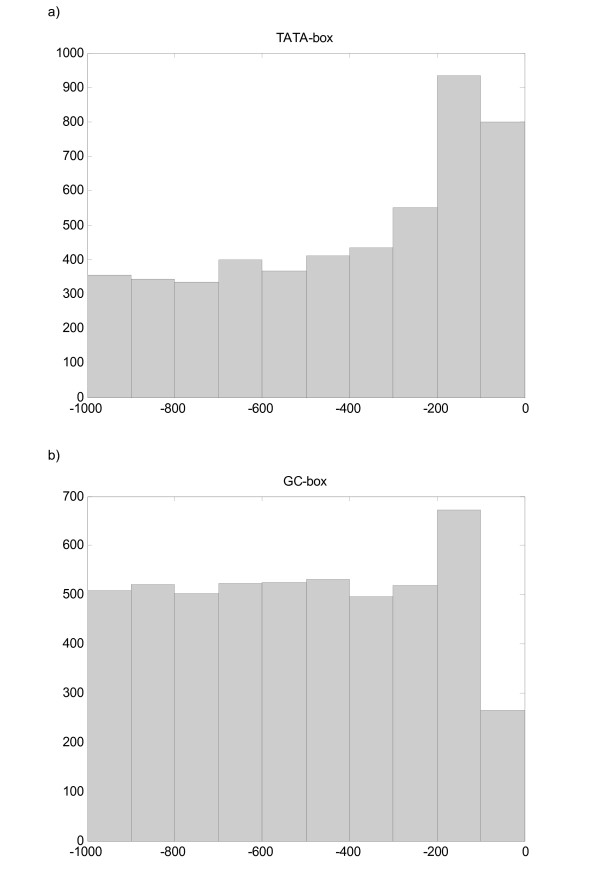
Positional distribution of specific motifs that correspond to *TATA-box *and the *GC-box *in the global *Saccharomyces cerevisiae *data set.

Figure 4(a) shows the positional distribution of the TATAAA motif, the most common consensus sequence for the *TATA-box *in the yeast *Saccharomyces cerevisiae *[18]. In this data set this motif is present in 3242 out of 5864 promoters. In metazoans, the *TATA-box *is typically located about 25 – 30 nt upstream of the transcription start site. However, in *Saccharomyces cerevisiae*, this box has a more variable position that ranges from about 40 to 100 nt upstream of the start site, and it has been observed that a wide range of sequences can function as a *TATA-box *in vivo [19].

In Figure 4(b) it is also possible to observe the positional distribution of a set of motifs that corresponds to the *GC-box*. The p-values obtained for the uniformity test for these elements are much lower than 0.05, suggesting non-uniformity and stressing that these motifs have a positional preference.

However, only the *TATA-box *has been classified as over-represented in a statistically significant way. This is a case where the application of a non-uniformity test would identify biologically significant motifs that would go unnoticed by the over-representation tests.

The results obtained for this data set show that most of the extracted motifs are classified as non-uniformly distributed. This result was somehow expected since the motif finder was instructed to extract motifs with a minimum quorum of 20% in a large data set of 5856 promoter sequences. This means that each motif must be present in at least 1172 sequences. Some of the motifs extracted correspond to known transcription factors binding sites and a large number corresponds to motifs that are important in the context of the chromatin regulation. Most of these motifs are known to have a positional preference. In this example, we did not perform a detailed analysis of all the motifs extracted, since they are too numerous. The objective was to identify well characterized motifs that have positional preference and that may not be over-represented in a statistically significant way.

### Aft2p

This data set contains the promoter regions of 46 genes that are documented to be regulated by the transcription factor *Aft2p*. The documented consensus for this TF are the motifs: CGCACCC, GGCACCC, TGCACCC and YKCACCCR.

For this data set, RISO was instructed to extract simple conserved motifs, with sizes between 5 and 8, and a minimum quorum of 50%. Table 2 presents the number of motifs classified in each of the four classes defined by the combination of the uniformity and over-representation statistical tests.

**Table 2:** Distribution of motifs according to uniformity and statistical significance in the *Aft2p *data set.

Motifs	Uniform	Non uniform	Total
Not over-represented	988	110	1098
Over-represented	15	10	25

Total	1003	120	1123

From a total of 1123 motifs extracted, only 120 are considered to be non-uniform. A set of 10 out of these 120 are classified as over-represented. This small group includes the motif CACCC, which corresponds to the core conserved motif described for this TF binding site. The p-value for the uniformity test for this motif was 1.75*e *- 4. The complete consensus that corresponds to this TF binding site was not reported, since this it is only present in a small number of sequences in this data set.

**Figure 5:**
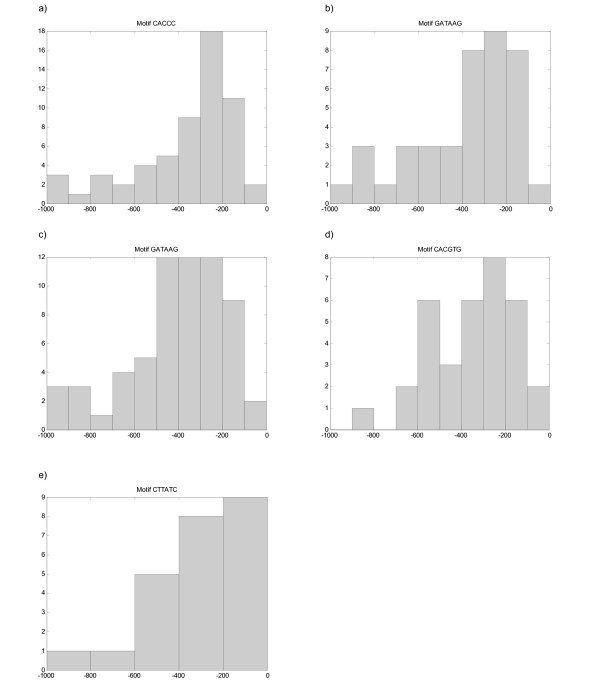
Positional distribution of the core motifs CACCC (a), GATAAG (b), GATAAG (c), CACGTG (d) and CTTATC (e) in the promoter sequences of the *Aft2p*, *Dal80p*, *Gln3p*, *Met4p *and *Gat1p *data sets, respectively.

Figure 5(a) shows the positional distribution of the conserved core motif, the motif CACCC, in the promoter region. It is possible to observe that this motif clearly has a preferential location.

### Dal80p

This data set includes 26 promoter sequences of genes documented to be regulated by the transcription factor *Dal80p*. The biological consensus described consists of two identical motifs, GATAAG, separated by 15 to 20 base pairs.

For this data set the motif finder was instructed to extract simple conserved motifs, with sizes between 5 and 6, and a minimum quorum of 50%. Table 3 presents the number of motifs included in each of the four classes.

**Table 3:** Distribution of motifs according to uniformity and 77 statistical significance in the *Dal80p *data set.

Motifs	Uniform	Non uniform	Total
Not over-represented	407	64	471
Over-represented	7	4	11

Total	414	68	482

From a total of 482 motifs, only 68 are distributed non-uniformly, and only 4 of these are over-represented in a statistically significant way. In this group of 4 motifs, it is possible to find the GATAAG motif, which is precisely the motif that corresponds to the biological consensus described. The three other motifs that complete this group are the motifs CTTATC, TATATA and AAGAAA. The first one is the complement of the GATAAG motif, the second one is a TATA-box that, as discussed before, has a positional preference and the last one is a motif already identified in yeast promoters as being one of the pre-mRNA 3'-end-processing signals.

Figure 5(b) shows the distribution of the GATAAG motif in the promoter region. This motif obtained a p-value of 0.04 in the uniformity test.

### Gln3p

This data set consists of 19 promoter sequences of genes regulated by the transcription factor *Gln3p*. The reported consensi for this TF binding site are the motifs GATAAG and GATTAG.

Motifs with sizes between 5 and 6 bases and a minimum quorum of 50% were extracted by the motif finder. Table 4 presents the number of motifs included in each of the four classes. A total of 746 motifs was extracted and classified. From these, only 175 show positional preference. In this group, 14 were classified as over-represented. In this last group it is possible to find the motif GATAAG, that corresponds to one of the consensi described as a binding site for this TF. The group also includes motifs that correspond to the general transcription factor *TATA-box*: TATATA, ATATAA and ATATAT and also the complement of the motif of interest. The second consensus, GATTAG, was not found in any of the groups. However, in the group of the 161 motifs that are not over-represented but are non-uniformly distributed, it is possible to find the motif CTAATC, that corresponds to the complement of this motif. In this case the non-uniformity test may play a key role, if used to distinguish this motif from background noise.

**Table 4:** Distribution of motifs according to uniformity and statistical significance in the *Gln3p *data set.

Motifs	Uniform	Non uniform	Total
Not over-represented	559	161	720
Over-represented	12	14	26

Total	571	175	746

Figure 5(c) shows the positional distribution of the GATAAG motif. The p-value obtained in the uniformity test was 0.02, which represents a strong indication of non-uniform distribution.

### Met4p

This data set contains 36 promoter sequences of genes that are documented to be regulated by the transcription factor *Met4p*. The documented consensus for this TF binding site is the motif TCACGTG. The motif finder was instructed to extract motifs with sizes between 6 and 7 bases and a minimum quorum of 50%. Table 5 presents the results. A total of 130 motifs was extracted with these characteristics. From these, only 13 were classified as over-represented and non-uniformly distributed. This set of motifs includes the motif CACGTG, the only one that matches the described consensus. The p-value obtained with the uniformity test for this motif was 7.6*e *- 4. As expected, motifs related to the *TATA-box*, like the ATATAA, ATATAT and TATATA, were also among this set of relevant motifs.

**Table 5:** Distribution of motifs according to uniformity and statistical significance in the *Met4p *data set.

Motifs	Uniform	Non uniform	Total
Not over-represented	83	15	98
Over-represented	17	15	32

Total	100	30	130

Figure 5(d) shows the positional distribution of the motif CACGTG in the promoter region of the genes in the data set.

### Gat1p

This data set consists of 19 promoter regions of genes that are regulated by the transcription factor *Gat1p*. The consensus sequence of interest is the motif GATAAG. Motifs with sizes between 5 and 6 nucleotides and with a minimum quorum of 50% were extracted. Table 6 shows the results. A total of 765 motifs were identified, with only 3 being classified as over-represented and non-uniformly distributed. The motif GATAAG is included in the group of motifs that were classified as non over-represented and uniformly distributed (the p-value for the uniformity test for this motif was 0.06, close to the limit value of 0.05). However, the motif that has been classified as the most over-represented and also non-uniformly distributed was the motif CTTATC, that is the complement of the motif of interest. Although, the motif GATAAG is reported as the binding site for the *Gat1p *transcription factor, its reverse can also be considered [20,21]. This reverse motif is particularly relevant if present between positions -238 and -233. This position preference is exactly what is observed in this data set. The two other motifs in this category are the motifs TATATA and AAGAAA, whose biological relevance was already reported in the analysis of the data set *Dal80p*.

**Table 6:** Distribution of motifs according to uniformity and statistical significance in the *Gat1p *data set.

Motifs	Uniform	Non uniform	Total
Not over-represented	682	75	757
Over-represented	5	3	8

Total	687	78	765

Figure 5(e) shows the positional distribution of the motif CTTATC in the promoter regions of the genes considered.

Datasets for other organisms have also been analyzed, and confirm the obtained results are consisten with the ones presented in this section [see Additional file 1].

## Conclusion

Given these results, we propose that the integration of position uniformity tests and over-representation tests can be used to improve the accuracy of the classification of motifs found by combinatorial motif finders.

The study we performed on the positional distribution of several well known cis-regulatory elements, in the promoter sequences of different organisms, has shown that position conservation is a significant characteristic of many biologically significant motifs. In particular, the results show that many biologically relevant motifs appear heterogeneously distributed in the promoter region of genes, and therefore, that non-uniformity is a good indicator of biological relevance.

In a number of instances where over-representation tests do not provide conclusive results, non-uniformity tests can be used to flag the relevant motifs. This effect is most evident for small motifs, that are expected to appear a large number of times, and for small datasets, where the over-representation tests are not powerful enough to single out motifs that are biologically significant, but are only somewhat over-represented.

In particular, it is clear from the results that motifs that pass both tests are very strong candidates for further analysis. This is an important point, specially for users of combinatorial motif finders, because it is sometimes hard to filter the relevant motifs from the large number of sequences identified by these methods. In fact, currently used over-representation tests are likely either to miss important motifs that are not long enough, or to flag as significant motifs that are over-represented only by chance.

## Methods

### Motif finding

Motifs were extracted using the motif-finding algorithm RISO [5]. This combinatorial method extracts motifs consisting of plain nucleotide sequences or sequences over a degenerate alphabet, explicitly enumerating all possible patterns. This approach has proved to be effective and efficient when the appropriate parameters for the extraction are known. In our case, since the motifs of interest were known, the parameters were selected to include the desired motifs, taking into account some degeneration of the patterns.

It is noteworthy that the application of the proposed approach is independent of the method used to model and extract the motifs. It could be used, for instance, on the list of occurrences reported by a probabilistic algorithm. Except for efficiency reasons, it could also be applied to test all possible oligonucleotide sequences without performing any searching and pre-filtering provided by the RISO combinatorial algorithm. This step is required only to reduce the number of tests to be performed and study only a subset of motifs that have particular properties. This can be used to specify features such as minimum quorum, length and degeneration level, guaranteeing the retrieval of all the motifs that fulfill them. Since this pre-selection could introduce a bias in the analysis, we subsequently studied motifs for which the biological relevance is widely reported.

### Over-representation tests

The statistical significance of over-representation of the extracted motifs was assessed using a model for the sequences based on a first-order Markov chain [22]. The probability of occurrence of a motif in each sequence is used to estimate the probability of the motif occurring in at least *k *sequences, by computing the distribution of a sum of Bernoulli variables, each one of them taking the value 1 in accordance with the computed probability for that sequence. This leads to a binomial distribution, if all sequences have equal length, or to a sum of unequal parameter Bernoulli random variables, if the lengths of the sequences are different.

The statistical significance of the observed number of occurrences for each motif is reported as a *p*-value, that is subsequently used to sort the output, starting with the most over-represented patterns. In this test, only the sequence of bases that constitutes the motif is considered and not the positions where the motif occurred in the sequences.

### Uniformity tests

The vector of occurrences **p **to be tested consists of the positions (*p*_1_, *p*_2_, ..., *p*_*n*_) where the target motif appeared (*n *times) in a set of *k *sequences {**S**_1_, **S**_2_, ..., **S**_*k*_}. The null hypothesis *H*_0 _is that the underlying distribution *F *of these positions is the discrete uniform *F *~ *Unif*{1, ..., *L*}, where *L *is the (common) length of each sequence **S**_*j*_.

In order to standardize the results across different sequences, the simulations were performed using a continuous uniform distribution *Unif*(0, 1). This corresponds to using vectors of the absolute position normalized over the total sequence length. This is equivalent to the discrete version if the continuous values are rounded or truncated to a finite number of classes. The results can be generally interpreted as relative positions to the beginning of transcription.

Uniformity tests abound in the literature. Some of the most widely used, and that will be applied in this study, are the Kolmogorov-Smirnov (KS) test and the Chi-Squared (CS) test.

The KS test is based on the comparison between the empirical distribution function and the cumulative distribution function specified by the null hypothesis. The test statistic *D *is given by:

(1) $$D=\underset{1\leq i\leq n}{\max}\left|F(Y_{i})-\frac{i}{n}\right|$$

where *n *is the sample size and *F *is the distribution function associated with the theoretical hypothesis. The null hypothesis is rejected if *D *is greater than a critical value which is dependent on the level of significance considered and the sample size.

The CS test is based on the difference between each observed and theoretical frequency for each possible outcome. The test statistics *X*^2 ^is calculated using the expression:

(2) $$X^{2}=\sum_{i=1}^{k}\frac{{\left(O_{i}-E_{i}\right)}^{2}}{E_{i}}$$

where *O*_*i *_is the observed frequency and *E*_*i *_is the expected (theoretical) frequency asserted by the null hypothesis. The test statistic *X*^2 ^follows approximately a chi-square distribution with (*k *- *c *+ 1) degrees of freedom, $\chi_{k-c+1}^{2}$, where *k *is the number of classes and *c *is the number of estimated parameters.

The CS tests were conducted by fixing the number of classes or bins for each sample in order to find, for each particular distribution, the most discriminative parameter set. Cochran'rule was used as the starting point to define the number of classes of equally spaced edges, by forcing a minimum number of five expected observations per bin. This corresponds to employing, for each sample size *N*, *c *= ⌊*N*/5⌋ classes. Other values were also tested to assess if this rule was adequate to this specific setting, specially for small samples where the chi-square approximation might not be applicable.

### Bootstrap analysis

Since the approximation to the Chi-square distribution described above is not accurate and might not be appropriate for small sample sizes, a bootstrap version of the Chi-square test was also implemented and analyzed. Parametric bootstrap methodologies are adequate to deal with few observations [15] and goodness-of-fit tests can be easily adapted to this methodology.

In parametric bootstrap the objective is to infer a characteristic *θ *from a sample (*x*_1_, *x*_2_, ..., *x*_*n*_) taken from a population with known distribution *F*. This method also applies to hypotheses tests: we have to establish the hypothesis *H*_0 _and *H*_1 _to test and then choose the appropriated statistic to discriminate between these hypotheses. After calculating the observed statistic *t*_*obs *_for the sample under analysis, *B *samples are generated from the distribution associated with *H*_0_. For each sample we then calculate the value *t*_*i *_of the test statistic, *i *= 1, ..., *B*. The *p*-value is subsequently obtained using the expression:

(3) $$p\text{-value}=\frac{\sum_{i=1}^{B}I(t_{i}\geq t_{obs})+1}{B+1}$$

where *I *is the indicator or characteristic function that counts the number of replicates whose *t*_*i *_values exceeded the original *t*_*obs*_.

*H*_0 _is accepted or rejected according to the significance level, *α*, established. For a fixed significance value, *α*, uniformity can be rejected if *p*-value ≤ *α*. The smaller the *p*-value, the more unlikely is that the sample came from a uniform distribution. Intuitively, if most of the *B *samples taken from a uniform distribution have a smaller value of the statistic than the original sample, then it is not likely that our initial sample comes from a uniform distribution.

Since the described bootstrap procedure should be done every time a uniformity test is run for a given sample, a pre-simulation was performed in order to calculate the bootstrap critical values, thus avoiding unnecessary simulations. Chi-square tests with varied sample sizes and number of classes were performed on uniformly generated samples and the results saved for future comparison. This procedure was conducted on 200000 replicates.

### Sensitivity, specificity and ROC curves

The sensitivity and specificity were calculated according to the expressions:

(4) $$sensitivity=\frac{TruePositives}{TruePositives+FalseNegatives}$$

(5) $$specificity=\frac{TrueNegatives}{TrueNegatives+FalsePositives}$$

where *Positives *are the samples from the Uniform distribution and *Negatives *are samples generated from an alternative distribution. In this study the Beta distribution with several parameters *Beta*(*α*, *β*) was considered, given its flexibility and properties. This distribution is defined on the (0, 1) interval, and if (*α*, *β*) = (1, 1), it reduces to the uniform distribution. For parameters (*α*, *α*) the function has a bell-shape and is symmetric and centered on 0.5. For distinct values of *α *and *β *it becomes asymmetric. This distribution exhibits a wide spectrum of behaviors and can therefore be used to model the positional distribution of motifs in sequences.

There is a close relationship between the previous definitions and hypothesis testing when the null hypothesis *H*_0 _is the uniform distribution of the positions. In this case, the level of significance *α*, defined as the probability of rejecting *H*_0_, when it is true, is related with the sensitivity of the test and equal to 1 - *sensitivity*. The power of the test, the probability of rejecting *H*_0 _when it is false, is equivalent to the specificity defined as above.

The level of significance used in the tests was 0.05, unless otherwise noted. The sensitivity and specificity of a test depend on the level of significance considered. For this reason *Receiver Operating Characteristic curves *(ROC curves) are also presented. ROC curves are graphical plots of the *sensitivity *versus 1 - specificity for several thresholds values, in this case the test statistics critical cutoff value. They allow the comparison between tests for different levels of significance.

### Datasets

To validate the proposed methodology, both artificially generated and real data sets were considered. The synthetic data sets correspond to vectors of motif positions generated from different distributions. Each data set consists of 40000 samples of different lengths (10, ... ..., 100) that were simulated from the following distributions:

1. Uniform continuous distribution on (0, 1) or, equivalently, *Beta*(1, 1)

2. *Beta*(*α*, *β*) distribution with parameters (*α*, *β*) equal to (3, 3), (15, 15) and (3, 1.5), reflecting different degrees of asymmetry.

The three tests referred (KS, CS and CS-boot) were then applied to each of the samples in order to obtain a mean rejection rate across all the replicates. These mean values estimate the sensitivity and specificity of the test of uniformity, or, equivalently its level of significance and power.

The advantage of using synthetic data sets is the ability to specify exactly the distributions of the motif positions. In this way, we can safely compare the tests under analysis and analyze and tune the parameters involved. However, these artificial samples might still be very different from real motif positions, since the distributions considered are probably not accurate enough to model the actual motif positions in promoter regions.

For the validation on real data we defined two different types of sets. The first collection consists of data sets with a large number of promoter sequences that were used in the analysis of the positional distribution of well characterized core promoter elements, like TATA-box and GC-box. These data sets were extracted from several databases for several organisms: *Homo sapiens*, *Drosophila melanogaster*, *Saccharomyces cerevisiae*, *Escherichia coli *and *dicot plants*. Table 7 presents details for each set, for all the organisms considered, and the database reference from where the sequences were collected.

**Table 7:** Data sets for the analysis of the positional distribution of well characterized core promoter elements.

Species	Database	Seq. number	Average bp	Reference
*Homo sapiens*	EPD	1871	600	[24]
*Drosophila melanogaster*	DCPD	205	92	[25]
*Saccharomyces cerevisiae*	YEASTRACT	5864	1000	[23]
*Escherichia coli*	RegulonDB	1103	80	[26]
Dicots	PlantProm	220	251	[27]

The second collection of data sets correspond to promoter sequences from genes that were documented to be regulated by specific transcription factors, for *S. cerevisiae*. This information was gathered from YEASTRACT [23], an information system of transcription regulatory associations in *S. cerevisiae*. The rational for the choice of the five transcription factors presented was the existence of a consensus sequence that was already validated as the transcription factor cis-regulatory binding site. Table 8 presents details for each set and the consensus sequences that were checked for non-uniformity of their positional distribution.

**Table 8:** Data sets for for the analysis of the positional distribution of specific transcription factors binding sites in *S. cerevisiae*.

Transcription Factor	Seq. number	Average bp	Consensus
Aft2p	46	1000	CGCACCC
			GGCACCC
			TGCACCC
			YKCACCCR
Dal80p	26	1000	GATAAGN{15,20}GATAAG
Gln3p	56	1000	GATAAG
			GATTAG
Met4p	36	1000	TCACGTG
Gat1p	19	1000	GATAAG

For all the real datasets, we used the promoter regions defined in each one of the databases from where the sequences were retrieved. For example, for the *S. cerevisiae *datasets the promoter region corresponds to 1000 nucleotides upstream of the translation start site.

## Authors' contributions

ACC developed the initial study, and implemented the initial versions of the statistical significance tests. SV participated in the design of the study and performed the statistical analyses. ATF contributed with the datasets and the analysis on biological data. All authors participated in the design of the experiments and cooperated in the writing of the final manuscript.

## Supplementary Material

Additional file 1 Analysis of datasets for other organisms. This file describes the results obtained in other organisms. Click here for file
