# Supplementary material for: An analysis of the positional distribution of DNA motifs in promoter regions and its biological relevance
Source: BMC Bioinformatics. 2008 Feb 7;9:89. doi: 10.1186/1471-2105-9-89 (PMC2375121; doi:10.1186/1471-2105-9-89)
Supplement: Additional file 1 — Analysis of datasets for other organisms. This file describes the results obtained in other organisms. [file 1471-2105-9-89-S1.pdf]

An analysis of the positional distribution of DNA motifs in promoter regions and its biological relevance: additional materials and results

Ana C. Casimiro[1], Susana Vinga[1,2], Ana T. Freitas[1], Arlindo L. Oliveira*[1]

[1]INESC-ID/IST, Rua Alves Redol, 9 1000-029 Lisboa, Portugal
[2]FCM/UNL, Campo dos Mártires da Pátria 130, 1169-056 Lisboa, Portugal

Email: Ana C. Casimiro - ana.casimiro@gmail.com; Susana Vinga - svinga@algos.inesc-id.pt; Ana T. Freitas - atf@inesc-id.pt; Arlindo L. Oliveira*- aml@inesc-id.pt;

*Corresponding author

Additional results on other organisms

This appendix includes the results obtained in other organisms, as referred in the main paper, namely *D.melanogaster*, *E.coli*, *H.sapiens*, *A.thaliana* and several Dicotyledons plants.

Drosophila

For this dataset there are reported three general factors: *TATA-box*, *DPE* (downstream promoter element) and *Iniciator*. The *TATA-box* consensus consists of a sequence with 5 of 6 nucleotides conforming to the consensus TATAWAWR. The *DPE* consensus consists of a sequence with 5 of 6 nucleotides conforming to the *DPE* functional range set A/G/T - C/G - A/T - C/T - A/C/G - C/T. The *Iniciator* is described by the range set t/g-C-A/t-g/t/c-t/c/a-c/t-t/c/g-t/c.

Figure 1 shows the distribution of the documented general transcription factors described for *Drosophila melanogaster*. The p-values obtained using the proposed test are respectively 9.999×10^{-5}, 0.0789 and 0.0789. Both histograms and numerical values suggest that these factors do not locate randomly along the promoter region but have positional preferences.

We extracted motifs with length between 5 and 7, with a minimum quorum of 20%. All the motifs were classified according to uniformity and over-representation as described above. Table shows the results obtained.

Table 1: Distribution of motifs according to uniformity and over-representation in promoters of *Drosophila melanogaster*.

motifs	non uniform	uniform	Total
significant	29	10	39
non significant	75	153	228
Total	104	163	267

From the total of 267 motifs only less than a half (104) have a non-uniform

distribution. In this group 29 have a high significance. Amongst these are present motifs that refer to the *TATA-box* (TATAT, TATATA, TAAAA and ATATAA are some good examples) and the *Iniciator* (for instance, TCAGTC, CAGTC, AGTTG and TCAGT). In the group of non-uniform but with non significance we can also find some motifs that refer to the Iniciator. There are no motifs in the non-uniform group that refer to the *DPE* element.

Since the documented elements *Iniciator* and *DPE* are flexible, it is possible to find motifs that refer to them in different groups. Therefore, we did another extraction of longer conserved motifs: sequences of 6 to 9 bases with minimal quorum of 10%. Table shows the results of this second procedure.

Table 2: Distribution of the 14 motifs with length between 6 and 9 for the *Drosophila melanogaster* promoters.

	non uniform		uniform
significant	AAAAGC	ATAAAA	GAAAAA
	ATAAAAG	ATATAA	
	ATCAGT	ATCGAT	
	TAAAG	TATAAA	
	TATAAAA	TATAAAAG	
	TATATA	TCAGTT	
non significant			AAAAAA

Given the higher restriction in the parameters, only 14 motifs were obtained, with 12 of these classified as non-uniform. In this group, only ATCGAT doesn't match with any of the described elements. The motifs AAAAGC, ATAAAAG, TAAAG, TATAAAA, TATATA, ATAAAA, ATATAA, TATAAA and TATAAAAG match the consensus for *TATA-box*, and ATCAGT and TCAGTT match the description for *Iniciator* element. The uniformly distributed motifs obtained do not match with any of the described elements. We did not find motifs that relate to the *DPE* element, maybe because it is not very conserved. The uniformly distributed motifs obtained, GAAAAA and AAAAAA, do not fit in any of the described elements.

E. coli

This dataset contains 1103 promoter regions from *E. coli*. For this organism there are documented three well conserved motifs: TTGACA (-35), TATAAT (-10) and AAAATTATTTT (-50 to 20). Figure 2 shows the positional distribution for the first two elements. These motifs both obtained the p-value 9.999×10^{-5} indicating that they don't locate uniformly along the promoter region.

We extracted motifs between 5 and 8 bases with minimum quorum 10%. Table shows the results. From the total of the 172 motifs, 120 do not distribute uniformly, and from these only 30 are considered strongly over-represented. In this group we found TATAA and ATAAT, which refer to the consensus TATAAT described. We also found AAAAT and AAAAAT, two motifs that can refer to the third consensus described (an A-T rich element). There are no other relevant motifs in this group. In the group of the 90 non-uniform and less significant motifs there are some relevant motifs: TTGAC is the only motif that relates to the second biological motif described; TTTTT, TTTTTTT, AATTA, TATTT and other similar motifs also refer to the A-T rich motif.

Table 3: Distribution of motifs according to uniformity and over-representation in dataset *E. coli*.

motifs	Uniform	Non-uniform	Total
Not over-represented	10	90	100
Over-represented	42	30	72
Total	52	120	172

In this dataset, there are some good examples of biological motifs that are not over-represented, but that have a positional preference, indicating that non-uniformity can help to correctly identify real motifs.

Dicot Plants

This dataset consists of 220 promoter regions of several dicot plants. The most relevant motifs are the TATA-box, the CAAT-box and the TSS (transcription start site). The documented profiles are given below:

- *TATA-box*:

C T A T A W A W A

- *CCAAT-box*:

n C A A T

- *TSS*:

W	n	T	C	A	w
		a	t	c	

Figure 3 shows the distribution of these elements along the promoter region. The correspondent p-values for each distribution were 9.999×10^{-5}, 0.751 and 1. The motif CAAT is a short motif that occurs commonly in the promoter region, and so, it is not considered to be uniformly distributed. The TSS profile allows some variation, it is not very well conserved. As a consequence, if we look up for all the motifs that agree with that profile, we get a collection of uniformly positions along the promoter region. The p-value obtained shows strong evidence of uniformity.

We extracted motifs between 4 and 6 bases with minimum quorum 30%. Table shows the results. From the total of 447 motifs, only 34 are over-represented and offer evidence of non-uniformity. Some of these motifs (13 in total) fit the *TATA-box* profile. The others don't relate to any of the mentioned profiles. The motif CCAAT was classified as non-uniform, but is not over-represented. There are many motifs that fit the *TSS* profile: they all got poor over-representation, some are considered uniform and others non-uniform. Weak conservation in the *TSS* profile may explain this result.

Arabidopsis thaliana

This dataset contains 1922 promoter sequences of the plant *Arabidopsis thaliana*. The relevant motifs are the ones described before for the dicot plants. Figure 4 focus the distribution of *Tata-box* and CAAT motif. The p-values obtained were 9.999×10^{-5} and 0.443 respectively.

Table 4: Distribution of motifs according to uniformity and over-representation in dataset Dicot Plants.

motifs	non uniform	uniform	Total
Not over-represented	185	187	372
Over-represented	11	34	45
Total	196	221	417

We did an extraction of motifs having 5 to 8 bases, with minimum quorum 50%. We obtained a total of 707 motifs, which are distributed according to table . From the 570 motifs that distribute uniformly, 139 are over-represented. In this group we found some motifs that fit the *TATA-box* profile (16 in total), and several motifs that relate to the *TSS* profile (45 in total). In the group of non-uniformly distributed motifs we can find some motifs that relate to the *TSS* element. In this group, we also find the motifs: CCAAT, ACAAT, GCAAT, TCAAT and other similar motifs that fit the *CAAT-box* profile.

Table 5: Distribution of motifs according to uniformity and over-representation in *Arabidopsis thaliana* dataset.

motifs	non uniform	uniform	Total
Not over-represented	185	187	372
Over-represented	11	34	45
Total	196	221	417

Homo sapiens

This dataset collects 1871 human promoter sequences. The general transcription factors described for eukariotic species are: *TATA-box*, *GC-box*, *CAAT-box* and the *Initiator Cap Signal*. The documented profiles are given above:

- *TATA-box*:

T	A	T	A	W	A	D	R

- *GC-box*:

g	G	G	G	C	G	G	g
c	a			t	a	t	a
a				a			t

- *CCAAT-box*:

A	g	C	C	A	a	T	c	A
g	a				t	a	g	g

- *Iniciator Cap Signal*:

t	C	A	g	t	c	t	t
g		t	t	c	t	c	c
			c	a		g	

Figure 5 shows the distribution of the profiles considered. All the elements got the same p-value: 9.999×10^{-5}, which is the smallest value that can be obtained according to the test used.

We extracted motifs between 5 to 8 bases with minimum quorum 50%. The total of 702 motifs obtained were classified and table shows the results. In the total of 104 motifs considered non-uniformly distributed and statisticaly significant we found motifs that fit three of the four profiles considered. ATAAA, TAAAA, TAAAT, AAATA and AATAA fit the *TATA-box* profile; GGCGG, AGGCG, GGCGGG and GGCGG relate to the *GC-box*, and finally CCATCAG and CTCAG fit the *Initiator* profile. Among the group of the 518 motifs we also found motifs that relate to the *CAAT-box*: CCAAG, GCCAA, CCAAA, ACCAA and CCAAT. In this group there are also motifs that correspond to the *Initiator* profile.

Table 6: Distribution of motifs according to uniformity and over-representation in *Homo sapiens* dataset.

motifs	non uniform	uniform	Total
Not over-represented	63	518	581
Over-represented	17	104	121
Total	80	622	702

In a general way, profiles that are not too conserved have many motifs that fit the profile, and as a consequence, we can find these motifs in very different groups. One of the most interesting situations is when the conserved part of the profile is in the non-uniform group and over-represented and the less conserved motifs are also classified as non-uniform but as less statistically significant. This reveals that, even if the profile is not very conserved, its location along the promoter region is!

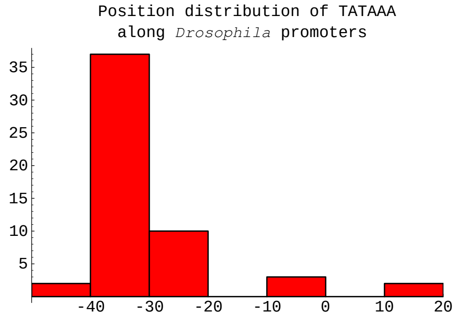

(a) *TATA-box*

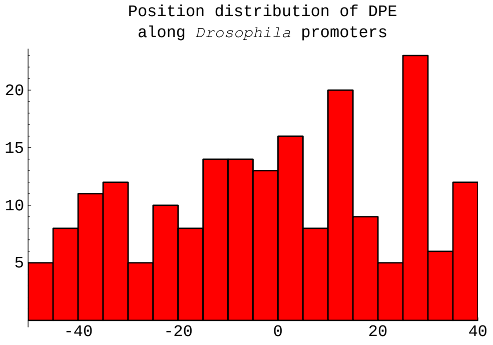

(b) *DPE-box*

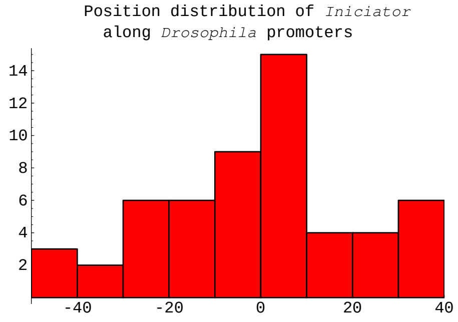

(c) *Iniciator*

Figure 1: Positional distribution of canonical transcription factors documented for *Drosophila melanogaster*.

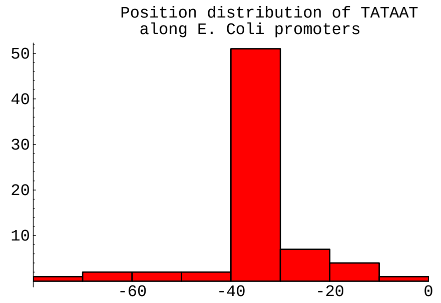

(a) *TATA-box*

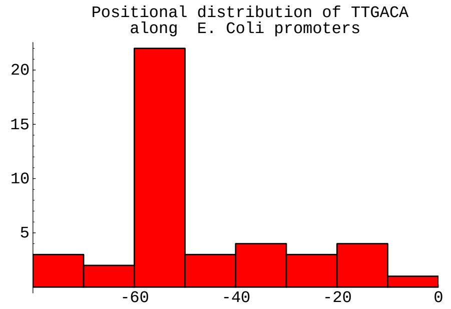

(b) *TGACA*

Figure 2: Positional distribution of canonical transcription factors documented for *E. coli*.

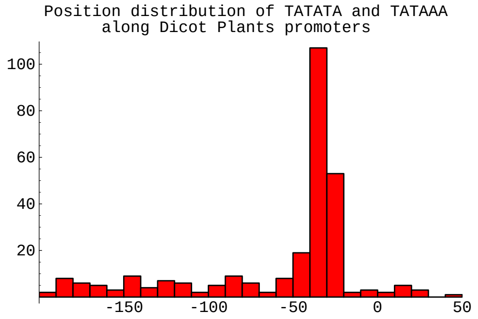

(a) *TATA-box*

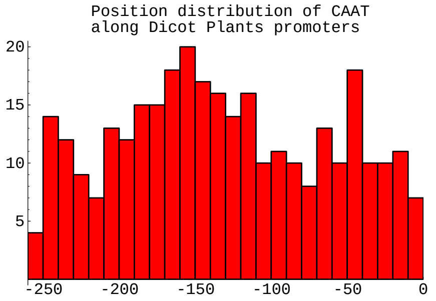

(b) *CAAT*

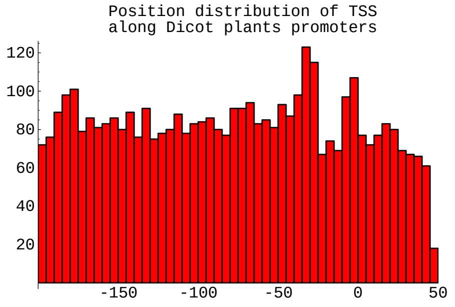

(c) *TSS*

Figure 3: Positional distribution of canonical transcription factors documented for *Dicot Plants*.

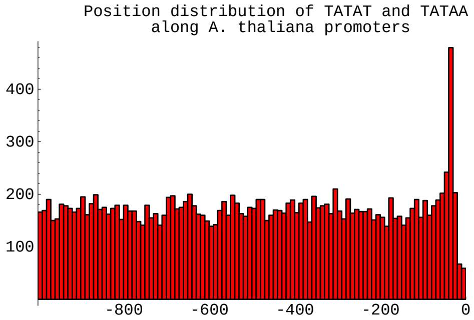

(a) *TATA-box*

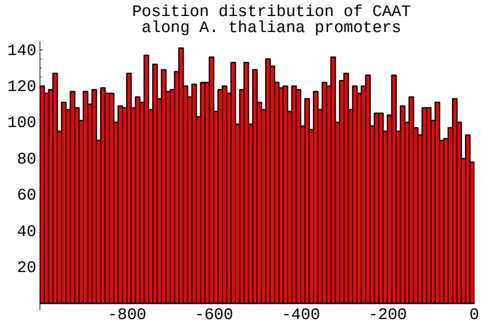

(b) *CAAT*

Figure 4: Positional distribution of *TATA-box* and CAAT for *Dicot Plants*.

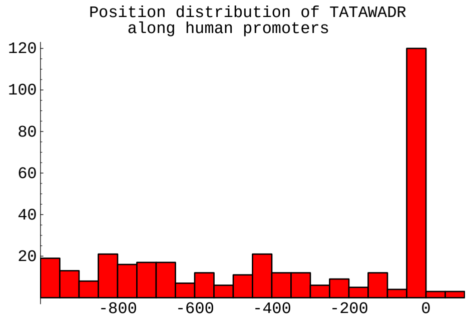

(a) *TATA-box*

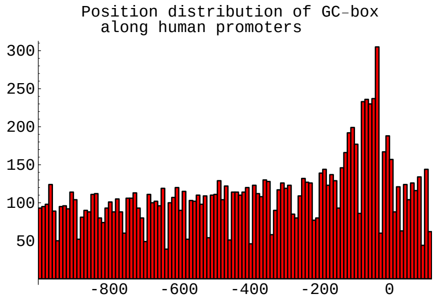

(b) *GC-box*

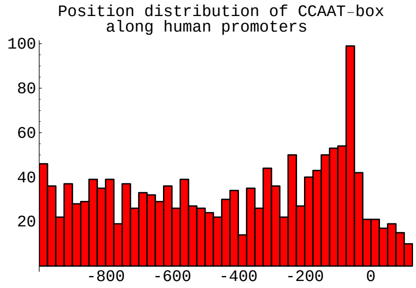

(c) *CAAT-box*

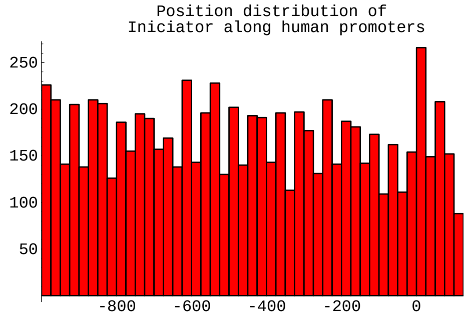

(d) *Iniciator*

Figure 5: Positional distribution of canonical transcription factors documented for humans.
